# Climate Changes, Natural Resources Depletion, COVID-19 Pandemic, and Russian-Ukrainian War: What Is the Impact on Habits Change and Mental Health?

**DOI:** 10.3390/ijerph191911929

**Published:** 2022-09-21

**Authors:** Benedetta Barchielli, Clarissa Cricenti, Francesca Gallè, Elita Anna Sabella, Fabrizio Liguori, Giovanna Da Molin, Giorgio Liguori, Giovanni Battista Orsi, Anna Maria Giannini, Stefano Ferracuti, Christian Napoli

**Affiliations:** 1 Department of Dynamic, Clinical Psychology and Health, “Sapienza” University of Rome, Via degli Apuli 1, 00185 Rome, Italy; 2 Department of Psychology, “Sapienza” University of Rome, Via dei Marsi 78, 00185 Rome, Italy; 3 Department of Movement Sciences and Wellbeing, University of Naples “Parthenope”, Via Medina 40, 80133 Naples, Italy; 4 Inter-University Research Centre “Population, Environment and Health”, University of Bari Aldo Moro, Piazza Cesare Battisti 1, 70122 Bari, Italy; 5 Family Psychotherapy Academy, Via Raffaele Morghen 181, 80129 Naples, Italy; 6 Department of Public Health and Infectious Diseases, “Sapienza” University of Rome, Piazzale Aldo Moro 5, 00185 Rome, Italy; 7 Department of Human Neuroscience, “Sapienza” University of Rome, Piazzale Aldo Moro, 5, 00185 Rome, Italy; 8 Department of Medical Surgical Sciences and Translational Medicine, “Sapienza” University of Rome, Via di Grottarossa 1035/1039, 00189 Rome, Italy

**Keywords:** Climate Changes, Natural Resources Depletion, COVID-19 pandemic, Russian-Ukrainian War, psychological well-being

## Abstract

Climate Change, Natural Resources Depletion, COVID-19, and Wars are some of the great challenges of our time. The consequences will affect psychological well-being and could have a harmful impact on mental health. This study aimed to assess the level of preoccupation and fears surrounding issues of the 21st-century and the implication for psychological well-being of the general population from Central/Southern Italy among different age groups. A questionnaire that included sociodemographic characteristics, topics formulated ad-hoc about preoccupation, fears, habits, and willingness to change habits in the future related to the 21st-century challenges, and the Depression Anxiety Stress Scales 21 (DASS-21) was administered online. A sample of 1831 participants (61% F; mean age 47.71 ± 17.30) was obtained. Results showed that young adults and older adults, respectively, reported greater and less psychological well-being. Young adults reported higher scores for preoccupation, changing habits, and willingness to change habits in the future, while older adults reported the lowest scores except for changing habits, also controlling for gender. Results for this variable, as well as correlations between the many variables described, rely on the specificity of age, and 21st-century challenges. Moreover, the main fears related to the 21st-century concerns were different based on both age and gender. In conclusion, the various stresses of the 21st-century discussed in this study have a relationship with personal well-being, and it is important to consider potential global mental health issues resulting from these stressors.

## 1. Introduction

The 21st-century brings many new and old challenges, and with them is an uncertainty that people need to overcome. Climate Change, Natural Resources Depletion, COVID-19, and Wars are all potential global health threats. Understanding how these concerns affect people’s well-being is a key research question.

### 1.1. Climate Change and Natural Resources Depletion

Within the last decade, the Italian government has focused on implementing national climate change adaptation strategies, mainly due to the consequences derived from this issue [1,2]. In fact, over time, there has been an increase in temperatures and a succession of extreme weather events, such as heavy rainfall [3] followed by long periods of drought [4], with effects, for example, on vegetation [5] or landslide risk [6]. However, alongside environmental problems, climate change also seems to be affecting the country’s economy, for example, due to the reduced attractiveness of Italy as a tourist destination [5].

The issue of climate change is usually addressed through environmental impact and its direct effects on humans, while there has been little effort to address the worry about future climate change and its effects on people’s mental well-being, especially the young. A large amount of research has focused on the impact of previous weather events and climate change-related natural disasters on mental health, with consequences on sleep, stress, and the development of anxiety, depression, and trauma-related disorders such as posttraumatic stress disorder [7]. Recently, new research observed the relationship between climate change awareness and concern and mental/emotional consequences, such as, people becoming anxious about their future and the future of the planet [8]. Eco-anxiety is a specific form of anxiety related to stress or discomfort caused by environmental changes and the awareness of them [9,10]. Eco-anxiety is a recently used term, although it is understudied and is an unfamiliar construct. The concept of eco-anxiety can include fear of an impending environmental catastrophe, anxiety associated with worsening environmental conditions, anxiety experienced about an ecological crisis, and distress experienced even when there is no immediate physical evidence or proximal physical evidence of climate change or climate-related threats to oneself [11,12]. A climate change risk perception model (CCRPM) has been proposed in the literature organizing risk predictors into four categories: cognitive, experiential, socio-cultural, and socio-demographic [13]. The work has been echoed by Bradley et al. [14], who highlight that climate change awareness seems to be necessary to contemplate risks, as well as sociodemographic variables (for example, gender, age, ethnicity, and perhaps even education level and income), are associated with distinctive patterns of climate change-related cognitions, attitudes, and behaviors. Eco-anxiety is an understandable reaction to the growing awareness of climate change and global problems resulting from ecosystem damage. A qualitative study conducted in the Italian context [15], showed a high general population’s climate change awareness, with higher scores in younger people. Younger people (18–39 years) are more concerned about the effects of climate change than older people (40–59 years; over 60), with greater concern for the impacts of climate change on the world’s population and the environment than for the impacts on themselves. In addition to age, gender also influences levels of knowledge and concern about climate change. In particular, females would appear to be more aware and more concerned than males [16,17]. These differences, however, are also related to other factors such as the economic development of the country in which people live. For example, in wealthier countries, women express more concern about climate change than males [17].

Some people may express high stress regarding climate change with associated psychological symptomatology. A study conducted in Italy [18], showed a relationship between eco-anxiety and anxiety and depressive symptoms, self-efficacy, awareness of environmental issues, and pro-environmental behaviors. Nevertheless, there may also be age differences in the effects of climate change on mental health. The climate change generation gap has been investigated because younger people have grown up with more exposure to the effects of global warming than their parents and grandparents [19]. However, the results are contrasting. Some studies have shown that young people are most concerned about climate change as they will experience the personal and social consequences of it [20], with an extensive data collection of 27,000 citizens in 28 European countries showing that those aged 15–24 express more concern about climate change than those aged 55 and over [21]. On the other hand, metanalysis showed that older individuals are concerned about climate and appear more likely to engage in behaviors to conserve natural resources and avoid environmental damage [22]. Although there are currently no specific studies on the topic, it is necessary to understand the long-term effects of eco-anxiety on mental health.

Natural Resources Depletion is causally connected to climate change [8]. In the 21st century, human consumption of the Earth’s resources has resulted in accelerating rates of air and water pollution, biological changes in the ecosystems, deforestation, land degradation, loss of biodiversity, and ultimately climate change [23,24]. To our best knowledge, there are no studies specifically investigating the perception of this phenomenon.

### 1.2. Worry about COVID-19

Currently, the world is facing the threat of the coronavirus pandemic caused by the newly developed and evolving SARS-CoV-2 virus, responsible for COVID-19 disease.

In the wake of the COVID-19 pandemic declaration, Italy has repeatedly applied severe restrictive measures related mainly to social distancing, even with almost total confinement of the population, with important individual, social and economic consequences still in place today [25].

As in other world countries [26,27,28], in Italy, during the COVID-19 pandemic, financial issues [29], fear of contagion and death [30,31], social isolation and lockdown [32] were the main concerns, with behavioral, physical and psychological effects resulting from mental health impairment [33,34]. Daily worries related to COVID-19 issues are concerning for the psychological maladjustment of the person, who must cope with stress related to various potentially dangerous conditions, with the likelihood of developing depression and/or anxiety symptoms [35,36,37]. In particular, women were more at risk for some clinical conditions impacting mental health [31,32,33,34,35,36,37,38,39,40]. Women were more fearful and concerned than males about COVID-19 [41]. Although men are more likely to experience negative health consequences, women are more concerned about such consequences, while appearing to be less concerned about the financial consequences of COVID-19 [42]. In addition, in Italy, maladaptive coping strategies such as rumination and catastrophizing are correlated with high levels of worry, which mediates the relationship between coping strategies and anxiety [43,44]. A further aspect regarding COVID concerns is the infodemic. The infodemic is an abundance of both true and false information that overwhelms the subject by taking away its ability to adequately process information [45]. COVID fear combined with the infodemic is associated with issues on a person’s well-being, such as eager use of social media and high psychological distress [46]. In Italy, the number of cases and deaths due to infection are reported daily, with the risk of increasing fear and panic in the population [25]. This is a vicious cycle, as overuse of social media can expose people to misinformation and misconceptions about COVID-19, increasing people’s uneasiness and eventually escalating to psychological distress [47].

In addition, restraint strategies have impacted various lifestyle habits, such as social relationships [48,49,50], mobility [51] or changes in physical activity, alcohol use, eating habits, smoking, and sleep [52,53,54,55,56,57], with implications for mental health [58,59,60].

In particular, a worrying increase in unhealthy behaviors in the elderly has been reported, with changes in sleep habits, PA, diet, and, consequently, weight [33].

The elderly have been one of the major targets of COVID-19 and its complications, but they have shown a satisfactory level of knowledge of COVID-19 and related control measures [33]. This could explain the lower levels of anxiety, depression, and stress observed in the older age groups [61,62]. This finding was also supported by a study conducted in Italy [63], that investigated the impact of COVID-19 on Subjective Cognitive Complaints (SCC) which appeared to be negatively related to physical and mental health [64]. Results showed that social isolation negatively affects both mental health and cognitive functioning (attention, temporal orientation, executive functions). However, being under 45 years old was found to be a significant risk factor for impairment in both cognition and mental health [63].

### 1.3. Russian-Ukrainian War

According to data reported by the Non-governmental Organization “Armed conflict location & event data project (Acled)” which specializes in conflict collection, analysis, and mapping, as of 21 March 2022, 59 armed conflicts were counted [65]. Studies in countries that experienced war and/or armed conflict showed a marked deterioration of mental health among the population directly involved [66,67]. There was risk of post-traumatic stress disorder, depression, anxiety, and other stress-related conditions [68], with a higher prevalence in women, associated with the fear of and experience of rape [69]. Since February 2022 in Europe, the armed conflict between Russia and Ukraine has been ongoing. Recent research investigated the mental health impact of war on civilians directly caught up in the Russian-Ukrainian war [70]. The results confirm the negative effects of war associated with mental health, fear, substance use, stress, loneliness, burnout, and other related conditions. To our best knowledge, there are no studies investigating psychological well-being about the extent of war in other parts of Europe in subjects not directly involved.

The world watches with concern and insecurity regarding the political, social, and economic consequences of the war. Many of those watching the outcome of the war express helplessness toward the situation. Although Italy is not a country directly involved in the ongoing war, its consequences also affect Italian citizens, along with the fear that the war may spread beyond its current borders. Italian citizens, as well as those in other European countries, are witnessing rising energy and other commodity prices and harrowing scenes through the media-bombing process, in which all news is focused only on the emergency theme. Prolonged or repeated exposure to helplessness, referred to as learned helplessness, is a risk factor for depression, particularly given the psychological sequelae of COVID-19 still present [71]. The influence of emotion regulation and emotional intelligence on aggressive policies toward the current Russian-Ukrainian War was investigated in an Italian sample [72]. The results showed the presence of negative emotions in the Italian population about a conflict in which they are not directly involved, such as anxiety, anger, and disgust. These findings are likely to have significant implications because the experience and expression of negative emotions could impact the population’s mental health [73,74,75].

### 1.4. Aims

The research purpose is to investigate 21st-century main concerns and related habit changes and mental health in a sample of people from Central and Southern Italy. The study goals are: (i) investigating the differences among groups in the highlighted variables; (ii) evaluating the relationship between 21st-century concerns and the population’s levels of psychological well-being.

## 2. Material and Methods

### 2.1. Procedures

This cross-sectional study was performed during the period March 2022–June 2022 involving over-18-year-old participants living in Central and Southern Italy. The recruitment was performed by an online survey sent through the main means of communication (i.e., mailing lists, instant messaging, WhatsApp) and social networks (i.e., Facebook, Instagram), to reach a large convenience sample. For this purpose, the mailing lists of WhatsApp/Facebook/Instagram groups/chats of university students, and people attending recreational, religious, and cultural associations were used. In most cases, a reference figure for both cultural and recreational associations was involved with the request of further sharing the questionnaire via their contacts, in order to reach the largest number of completed questionnaires. No payment was provided to those people who fulfilled the questionnaire. Inclusion criteria were: having reached the age of majority (18 years) and having electronic devices and an internet connection available to complete the questionnaire and the provision of informed consent. Expedited ethics approval was obtained from the Institutional Board of the Inter-University Research Centre “Population, environment and health” (CIRPAS), with the number 1303_2021.

### 2.2. Participants

A minimum sample size of at least 385 enrolled individuals would have been required to investigate the selected variables in the over-eighteen-year-old population in southern and central Italy (i.e., 21.229.663 residents over 18 years of age living in southern and central regions). The sample was calculated by a sample size calculator, based on the reference population, assuming a response proportion of 50%, a 95% confidence level, and a 5% margin error, as previously reported [33].

A total of 1831 participants joined the research, of these 39% identified as male (*n =* 715) and ages ranged from 18 to 93 years (M = 47.71; SD = 17.30). Overall, 63.1% (1156) and 36.9% (*n =* 675) were residents in Central and Southern Italy, respectively.

The sample was divided into four groups based on the age: young adults, aged 18 to 35 (*n =* 470; mean age = 24.18; SD = 4.81; 26.0% male); adults, aged 36 to 50 (*n =* 465; mean age = 43.29; SD = 3.89; 39.4% male); older adults, aged 51 to 60 (*n =* 460, mean age = 55.61; SD = 2.63; 50.9% male); elders, aged 61 to 93 (*n =* 436; mean age = 69.47; SD = 7.15; 40.4% male).

### 2.3. Materials

Apart from demographic characteristics, level of education, relationships, and work information were collected. The general characteristics of the four groups are detailed in Table 1.

Sixteen items, developed based on previous studies [33,52,56], were used to measure feelings experienced and perceived lifestyle changes about COVID-19 (4 items), Russian-Ukrainian War (4 items), Climate Change (4 items), and Natural Resource Depletion (4 items). In order to ensure content validity, the survey was reviewed by a panel of experts in the specific fields of health behavior, mental health, public health epidemiology, and clinical psychology. The survey was then revised according to their suggestions.

Specifically, the following were analyzed for each context: the main fear related to each 21st-century concern fear (for example, “Regarding the depletion of the planet’s resources, what do you fear most, for yourself or for your loved ones?”), the perceived level of preoccupation (for example, “Regarding Climate Change, how concerned are you?”), the perceived level of changing one’s habits as a result of the event or situation (for example, “Regarding the COVID-19 pandemic, how much did your habits change to reduce the risk of infection (e.g., always wearing a mask, avoiding contact with other people)?”), and the level of willingness to change one’s habits in the future in response to the event or situation (for example, “Regarding Russia-Ukraine war, how willing would you be to change your habits (e.g., send goods or money to the affected populations, limit the use of gas) in the future?”). Preoccupation, change habits, and willingness to change habits in the future items were evaluated on a 5-point Likert scale, from “1” (not at all) to “5” (very much). “Fear” items involved a multiple-choice response.

Mental health was measured using the Depression, Anxiety and Stress Scale–21 items (DASS-21) [76] which contained 21 items and three self-report scales designed to measure the emotional states of depression, anxiety, and stress. All subscales were rated on a four-point Likert scale ranging from 0 (never) to 3 (almost always). DASS-21 outcome scores were classified into three ranges: average, high, and extremely high. The DASS-21 obtained high reliabilities in the Italian validation study, with Cronbach’s alphas of 0.81, 0.88, and 0.94 for the Anxiety, Depression, and Stress subscales, respectively. In the Lovibond and Lovibond version of the DASS-21 [77], the subscales were scored as follows: normal (0–9), mild (10–12), moderate (13–20), severe (21–27), and extremely severe (28–42) for Depression; normal (0–6), mild (7–9), moderate (10–14), severe (15–19), and extremely severe (20–42) for Anxiety; and normal (0–10), mild (11–18), moderate (19–26), severe (27–34), and extremely severe (35–42) for Stress.

### 2.4. Data Analysis

Statistical analysis was conducted using SPSS (Statistical Package for Social Science; version 27.0; IMB SPSS; Armonk, NY, USA). First, descriptive analyses of whole sample characteristics were performed. Secondly, descriptive analyses concerning COVID, psychopathological symptoms and main fear related to 21st-century concern in the four groups were performed. A correlation analysis was performed between the psychopathological symptoms, COVID experience, preoccupation, and habit variables.

Finally, after verifying for normality, ANOVA was used to compare age groups differences on the preoccupation, and habits change, also controlling for gender. Statistical significance in the post-hoc analysis was determined using Bonferroni correction and defined at *p* < 0.05.

## 3. Results

### 3.1. Differences among Age Groups

This section contains the differences between age groups in the DASS-21 dimensions, main fears, perceived level of preoccupation, perceived level of changing one’s habits, and perceived level of willingness to change one’s habits in the future about to “21st century concern”. Table 2 shows differences between age groups and gender in main fears reported regarding 21th century concern.

Concerning the main fears related to COVID-19, fear about the possible vaccine consequences was the most frequent in older adults, compared with all other groups, while the lowest frequency was found in young adults. This fear was more frequent in women, among young adults and adults. Fear of disease and its consequences and likelihood of isolation was more frequent in young adults than in all other age groups, while the lowest frequency was found in older adults. Females reported more fear of the likelihood of being isolated in adults and elders groups, while reporting more fear of disease in all groups except in the older adults group.

Concerning the fear of Natural Resource Depletion, fear of rising prices of food products was reported more frequently among older adults than young adults. In the adults and older adults groups, the fear was higher in men, while it was reported more frequently by women, among young adults. Both the fear of likelihood of starvation and dying were reported more by young adults than by the other age groups. However, fear of likelihood of starvation was reported more by men among older adults, and fear of likelihood of dying was reported more by women in adults and elders groups. Fear of the rising cost of energy was more common among elders and less frequent in the young adult group. Gender differences showed higher frequencies in women among young adults and men among older adults.

Regarding the Russia-Ukrainian War, results showed no significant age differences in the fear of rising prices of food products; however, on the same topic gender differences were found, with women reporting more fears than men among adults and less among elders. Fear of the likelihood of starvation was more common among adults than among young adults. Only in the latter group was a gender difference found, with women being more frequent. Fear of the rising cost of energy and the likelihood of being isolated was reported more among older adults, with higher frequencies in men for both main fears, while lower fear was reported among young adults. Only in the young adult group, did women report a higher frequency of fear of the likelihood of being isolated. Lastly, fear of the likelihood of dying and the likelihood of the COVID-19 pandemic not ending or worsening due to war were reported more among young adults and less among older adults. Gender differences were found only for fear of the likelihood of dying, with a higher prevalence in women than men in all age groups.

The fear of the likelihood of being isolated and starving due to climate change was reported more by elders than young adults, while results showed no gender differences in all age groups. Fear of a rise in food prices was more common in the older adults group than in the young adults and adults groups. Women reported higher frequency among young adults and elders, while men were among the other age groups. Lastly, the fear of being forced to leave one’s home and dying was reported more by young adults than by other age groups. Compared to men, women reported a fear of the likelihood of being forced to leave their place of living and the likelihood of dying more frequently among young adults and older adults, and among adults and elders, respectively.

**Figure 1:**
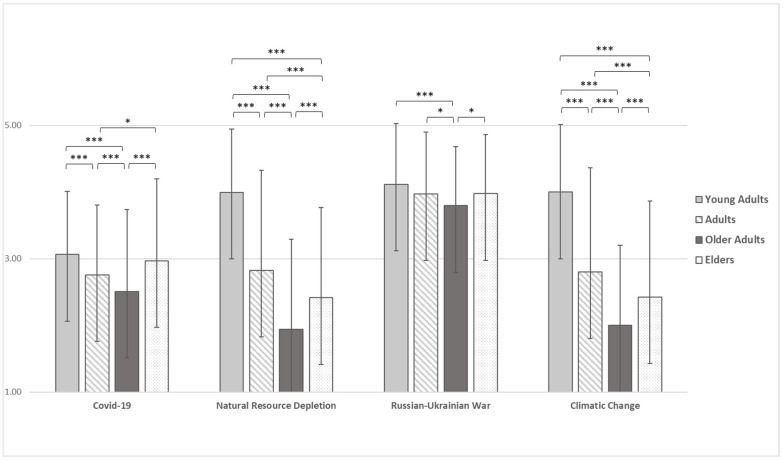
Differences among age groups in COVID-19, Natural Resources Depletion, Russia-Ukraine War, and Climatic Change preoccupation. Note. * = *p* < 0.05, *** = *p* < 0.001.

Figure 1 shows differences among age groups that emerged in perceived level of preoccupation with COVID-19, Natural Resources Depletion, the Russia-Ukraine War, and Climatic Change.

Results showed statistically significant differences in preoccupation with COVID-19 (F(3) = 22.986, *p* < 0.001), Natural Resources Depletion (F(3) = 224.552, *p* < 0.001), Russian-Ukrainian War (F(3) = 9.826, *p* < 0.001), and Climatic Change (F(3) = 198.644, *p* < 0.001) among age groups, also after controlling for gender (Table S1). About COVID-19 preoccupation, young adults reported a significantly higher mean (M = 3.06, SD = 0.94) than adults (M = 2.76, SD = 1.05) and older adults (M = 2.51, SD = 1.19), adults reported a significantly higher mean than older adults, and elders (M = 2.97, SD = 1.23) reported higher mean than both adults and older adults. Concerning both Natural Resources Depletion and Climatic Change the results showed a significantly higher mean in young adults (M = 4.00, SD = 0.95 and M = 4.00, SD = 1.01, respectively) than adults (M = 2.83, SD = 1.50 and M = 2.80, SD = 1.56, respectively), older adults (M = 1.94, SD = 1.20 and M = 2.00, SD = 1.2, respectively) and elders (M = 2.41, SD = 1.35 and M = 2.43, SD = 1.44) and adults reported higher mean than older adults and elders, and older adults showed a significantly lower mean than both adults and elders. Finally, about Russian-Ukrainian War preoccupation, although older adults reported a significantly lower mean (M = 3.80, SD = 0.93) than young adults (M = 4.12, SD = 0.91), adults (M = 3.97, SD = 0.90) and elders (M = 3.97, SD = 0.89); when controlling for gender, results show a significant difference only between older adults and young adults.

**Figure 2:**
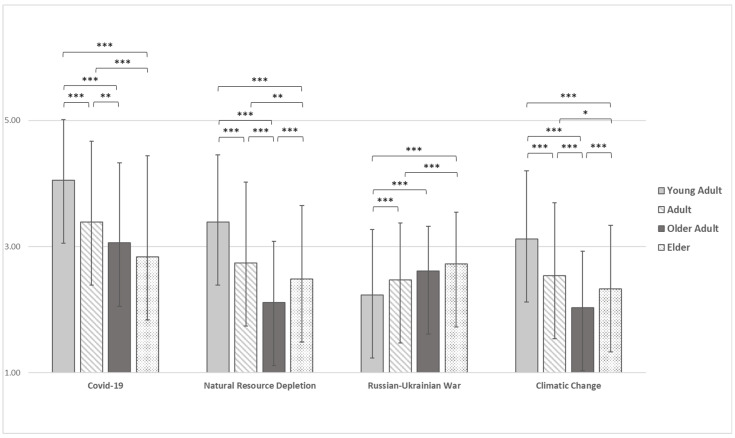
Differences among age groups for COVID-19, Natural Resources Depletion, Russia-Ukraine War, and Climatic Change related habits changes. Note. * = *p* < 0.05, ** = *p* < 0.01, *** = *p* < 0.001.

Regarding the perceived level of change in one’s habits as a result of the events (i.e., COVID-19 and Russian-Ukrainian War) and situations (i.e., Natural Resource Depletion and Climate Change) investigated, the differences among the age groups are shown in Figure 2.

Specifically, results showed statistically significant differences in habits change as a result of COVID-19 (F(3) = 65.766, *p* < 0.001), Natural Resources Depletion (F(3) = 86.999, *p* < 0.001), Russian-Ukrainian War (F(3) = 29.768, *p* < 0.001), and Climatic Change (F(3) = 76.265, *p* < 0.001), also after controlling for gender (Table S2). With regard to COVID-19, Natural Resources Depletion and Climatic Change, young adults reported a significantly higher mean (M = 4.05, SD = 0.96, M = 3.39, SD = 1.07 and M = 3.12, SD = 1.08 respectively) than adults (M = 3.39, SD = 1.28, M = 2.74, SD = 0.90 and M = 2.54, SD = 1.15 respectively), older adults (M = 3.06, SD = 1.27, M = 2.12, SD = 0.97 and M = 2.03, SD = 0.89 respectively) and elders (M = 2.84, SD = 1.60, M = 2.48, SD = 1.16 and M = 2.33, SD = 1.01). Results showed an opposite pattern about habits change as a result of Russian-Ukrainian War: young adults reported lower mean (M = 2.23, SD = 1.04) than other groups (adults: M = 2.47, SD = 0.90; older adults: M = 2.61, SD = 0.71; elders: M = 2.73, SD = 0.82). Moreover, adults reported higher mean than older adults and elder on habit change in response to COVID-19, Natural Resources Depletion and Climatic Change, while adults reported lower mean only than elders on Russian-Ukrainian War related habits change. Finally, concerning habits change as a result of Natural Resources Depletion and Climatic Change, elders reported higher mean than older adults.

In addition to the differences previously highlighted, when controlling for gender older adults reported significantly higher mean than elders in COVID-19 change habits and higher than adults in Russia-Ukrainian War change habits.

**Figure 3:**
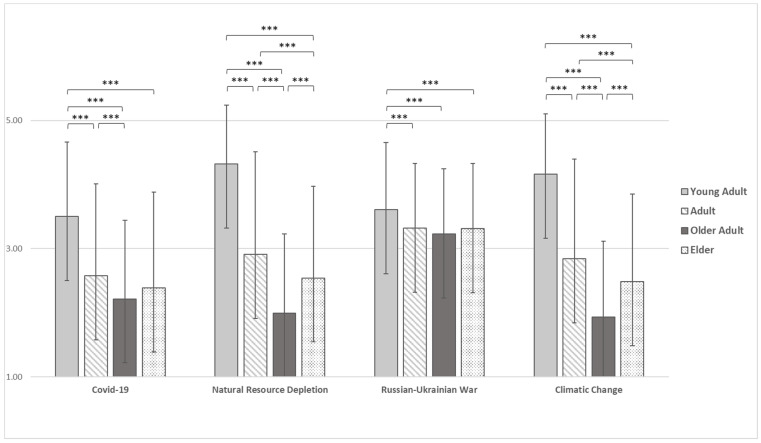
Differences among age groups in COVID-19, Natural Resources Depletion, Russia-Ukraine War, and Climatic Change Willingness to change one’s habits. Note. *** = *p* < 0.001.

Finally, differences among age groups on the perceived level of willingness to change one’s habits in the future in response to events (e.g., COVID-19 and the Russian-Ukrainian War) and situations (e.g., Natural Resource Depletion and Climate Change) explored are reported in Figure 3.

Statistically significant results emerged in the willingness to change one’s habits in the future in response to COVID-19 (F(3) = 85.927, *p* < 0.001), Natural Resources Depletion (F(3) = 263.475, *p* < 0.001), Russian-Ukrainian War (F(3) 12.254, *p* < 0.001), and Climatic Change (F(3) = 255.185, *p* < 0.001), also controlling for gender (Table S3). In all cases, young adults reported a significantly higher mean (M = 3.50, SD = 1.16, M = 4.32, SD = 0.92, M = 3.61, SD = 1.05 and M = 4.16, SD = 0.94, respectively) than adults (M = 2.58, SD = 1.44, M = 2.91, SD = 160, M = 3.32, SD = 1.01 and M = 2.84, SD = 1.56, respectively), older adults (M = 2.22, SD = 1.23, M = 1.99, SD = 1.24, M = 3.23, SD = 1.02 and M = 1.93, SD = 1.19, respectively) and elders (M = 2.39, SD = 1.50, M = 2.54, SD = 1.43, M = 3.31, SD = 1.02 and M = 2.49, SD = 1.36, respectively). Concerning the willingness to change one’s habits in the future in response to COVID-19, Natural Resources Depletion and Climatic Change, adults reported a higher mean than older adults, and about Natural Resources Depletion and Climatic Change adults report a higher mean also than elders. Finally, concerning the willingness to change one’s habits in the future in response to Natural Resources Depletion and Climatic Change, elders reported a higher mean than older adults. The differences between age groups in all dimensions of the DASS-21 are reported in the Supplementary Material (Figure S1), also controlling for gender (Table S4). Statistically significant results emerged on depression (F(3) = 72.879, *p* < 0.001), anxiety (F(3) = 54.469, *p* < 0.001) and stress (F(3) = 96.168, *p* < 0.001), also after controlling for gender (Table 3). In all dimensions, young adults reported a significantly higher mean (M = 12,98, SD = 10.17; M = 8.47, SD = 8.37; M = 17.23, SD = 10.14, respectively) than adults (M =7.41, SD = 7.01; M = 4.71, SD = 5.33; M = 10.83, SD = 8.49, respectively), older adults (M = 6.67, SD = 6.27; M = 4.30, SD = 4.43; M = 9.11, SD = 7.50, respectively) and elders (M = 6.67, SD = 6.66; M = 4.21, SD = 4.82; M = 8.99, SD = 7.67, respectively). With respect to the stress dimension, adults reported a significantly higher mean than older adults and elders. However, when controlling for gender, there is no significant difference between adults and older adults in the stress dimension.

### 3.2. Correlation Analysis

The correlations between depression, anxiety, stress, and perceived level of preoccupation and habit challenge in response to 21st-century challenges, for each age group are reported in Table 3.

**Table 3:** Correlation coefficients (Pearson’s r) between the DASS-21 and preoccupation and habits change scores.

		Cli_Pr	Res_Pr	Cov_Pr	War_Pr	Cli_ Hab	Res_ Hab	Cov_ Hab	War_Hab	Cli_F_Hab	Res_F_Hab	Cov_F_Hab	War_F_Hab
DASS_D	YoungAdults	0.224 **	0.182 **	0.096 *	0.175 **	0.078	0.115 *	0.169 **	0.019	0.182 **	0.167 **	0.131 **	0.131 **
	Adults	0.242 **	0.248 **	0.257 **	0.176 **	0.206 **	0.186 **	0.315 **	0.004	0.212 **	0.218 **	0.218 **	0.031
	Older Adults	0.100 *	0.057	0.230**	0.385 **	0.020	0.045	0.477 **	0.163 **	0.072	0.066	0.446 **	0.088
	Elders	0.172 **	0.173 **	0.232 **	0.471 **	0.106 *	0.142 **	0.339 **	0.069	0.128 **	0.145 **	0.275 **	0.147 **
DASS_A	YoungAdults	0.171 **	0.152 **	0.222 **	0.180 **	0.046	0.047	0.166 **	0.076	0.150 **	0.094 *	0.161 **	0.152 **
	Adults	0.225 **	0.230 **	0.262 **	0.158 **	0.157 **	0.148 **	0.310 **	0.024	0.199 **	0.181 **	0.267 **	0.091
	Older Adults	0.076	0.037	0.212 **	0.421 **	−0.020	0.000	0.486 **	0.221 **	0.078	0.046	0.438 **	0.109 *
	Elder	0.016	0.027	0.129 **	0.407 **	0.051	0.044	0.237 **	0.133 **	0.024	0.039	0.195 **	0.151 **
DASS_S	YoungAdults	0.150 **	0.161 **	0.144 **	0.215 **	0.063	0.088	0.249 **	0.031	0.213 **	0.173 **	0.154 **	0.149 **
	Adults	0.391 **	0.391 **	0.271 **	0.327 **	0.279 **	0.305 **	0.477 **	−0.035	0.362 **	0.373 **	0.371 **	0.135 **
	Older Adults	0.176 **	0.110 *	0.239 **	0.456 **	0.084	0.107 *	0.572 **	0.211 **	0.159 **	0.162 **	0.534 **	0.151 **
	Elder	0.112 *	0.125 **	0.192 **	0.525 **	0.067	0.112 *	0.357 **	0.078	0.088	0.114*	0.274 **	0.144 **

Abbreviations: Cli_Pr = Climatic Change preoccupation; Res_Pr = Natural Resources Depletion preoccupation; Cov_Pr = COVID-19 preoccupation; War_Pr = Russia-Ukraine War preoccupation; Cli_Hab = Climatic Change Habits; Res_Hab = Natural Resources Depletion Habits; Cov_Hab = COVID-19 Habits; War_Habit = Russia-Ukraine War Habits; Cli_F_Hab = Climatic Change Future Habits; Res_F_Hab = Natural Resources Depletion Future Habits; Cov_F_Hab = COVID-19 Future Habits; War_F_Hab = Russia-Ukraine War Future Habits. * *p* < 0.05; ** *p* < 0.01.

Findings showed that the COVID-19 and Russian-Ukrainian War preoccupations were significantly and positively correlated with Depression, Anxiety, and Stress severity in all groups. Both Natural Resources Depletion and Climatic Change preoccupations were significantly and positively correlated with stress in all groups and anxiety only in young adults and adults. Concerning Depression, there was a significant and positive correlation between Natural Resources Depletion in all groups except older adults and Climatic Change in all groups. With regard to the change of habits, Pearson’s correlation analysis (Table 3) showed that the past and future habit changes due to COVID-19 were significantly and positively correlated with Depression, Anxiety, and Stress in all groups. Past habit changes due to the Russian-Ukrainian War were significantly and positively correlated with Depression only in older adults, Anxiety only in adults, and Stress in all groups except young adults. Willingness to change habits in the future due to the Russian-Ukrainian War was significantly and positively correlated with Depression in young adults and elders, Anxiety in all groups except adults, and Stress in all groups. Past and future habit changes due to the Natural Resources Depletion were significantly and positively correlated with Depression in all groups except older adults, with Anxiety only in adults and in young adults and adults, respectively, and with Stress in all groups except young adults and all groups, respectively. Finally, past and future habit changes due to the Climatic Change were significantly and positively correlated with Depression in adults and elders and all groups except older adults, respectively, Anxiety in adults and young adults and adults, respectively, and Stress, in adults and all groups except elders, respectively.

## 4. Discussion

With reference to the goals of the study, firstly, the perception of worries about phenomena we called “21st-century concerns” was investigated across different age groups. The results showed differences in preoccupation with COVID-19, Natural Resources Depletion, Russian-Ukrainian War, and Climatic Change among the different groups. Young adults would seem to be the most concerned group in all categories, while older adults are the least concerned. Second, the relationship between the concerns of the 21st century and the population’s levels of psychological well-being was investigated. The results showed an association between the variables of anxiety, depression, stress, and the concerns of the 21st century, with specific differences in the groups.

Regarding preoccupation with Climate Change, previous studies demonstrated that young adults and older adults showed higher levels of preoccupation [19,20,21]. Our results show that younger adults are the most concerned compared to all groups, both in Climate Change and Natural Resources Depletion, but also older adults scored high, probably because Climate Change concerns is involving all age groups [19,22]. The motivations underlying the fear of climate change were found to be different among the groups, with young adults, older adults, and elders reporting more fear of being forced to leave their living place or dying, the rising food prices, and the likelihood of being isolated or starving, respectively. In contrast, concerning fear of Natural Resource Depletion, elders reported more fear of the rising cost of energy, while young adults reported more fear of starvation, in addition to the fear of dying. Interestingly, previous research showed that young adults are concerned about the consequences more frequently for the world’s general population and the environment than for themselves [15]. Future studies should further explore this issue to understand the different reactions in different age groups. Regarding gender differences, previous studies showed that women are more concerned about climate change [16,17]. Our results support previous findings by showing that gender differences in fear of Climate Change are age and main fear specific. For example, women reported more fear of rising prices of food products and energy among young adults, while men reported more fear of the likelihood of starvation from climate change among older adults. Finally, in older adults, Climate Change preoccupation would not appear to be associated with anxious symptomatology, in contrast to the younger groups, the only group reporting this relationship. Instead, depressive and stressful symptomatology is associated with preoccupation in all groups. Interestingly, regarding depletion of resources, the concern showed a relationship with all variables investigated by DASS, in the youngest and oldest groups, respectively. Previous studies have focused primarily on young adults and adolescents, showing that climate anxiety and other distressing emotions and thoughts about Climate Change impact their daily lives, similarly to our findings [9,18,78]. It is possible that young adults, compared to other age groups, are thinking more about plans for the future, which would explain the result regarding anxious symptomatology [9]. Further research, however, should investigate concerns about Climate Change in all age groups.

The young adults’ group had higher scores in preoccupation, change of habits, and willingness to change habits in the future linked to COVID-19. Older adults are the least worried, they express less fear, and seem to be less willing to change their habits in the future while elders are the group that has least changed their current habits. These results could be related to the previously demonstrated high level of COVID-19 knowledge and containment measures, which could decrease concern about the infection [33]. It should also be noted that while the elderly are one of the populations most vulnerable to the disease, they have also been the most successful in limiting social contact. Interestingly, our results showed that the fear of possible vaccine consequences was reported more by older adults, while fear of disease, its consequences, and the likelihood of isolation was reported most by young adults compared to elders. As also seen for climate change, this result could be related to a greater knowledge regarding COVID-19 and related to preventive measures among elders [33], which could explain less concern. At the same time, the importance of social connections among young people must also be taken into account, explaining why young adults experienced isolation with more concern and negative effects on mental health [49,50]. Consistent with findings from previous studies [41,42], women reported greater fear of disease and its consequences, but not in the elders group. Again, this could be explained by the lower concern for the elderly, regardless of gender.

As in other studies, the present study showed that older adults and elders presented with lower levels of anxiety, depression, and stress than younger adults during the COVID-19 outbreak [61,62]. Preoccupation with COVID-19 is related to all variables investigated by DASS in all groups while fear is related to depression and stress for adults and only stress in young adults. Our results correspond with studies showing the association between COVID-19 concerns and individual distress [35,39].

Changing habits and changing them in the future is related to anxiety, stress, and depression in all groups, according to results showing that people who experienced negative changes in lifestyle due to COVID-19 exhibited more psychological distress than people who did not experience such changes [58]. In particular, people who experienced lifestyle changes, due to COVID-19, may have used anxiety as their main emotional response to preventive measures to contrast COVID-19 spread [59] and the role of stress and anger in managing social distancing or fear of infection [60]. Finally, the association between habit change and mental health impairment could explain the lower willingness to change habits among the elderly who report lower mental health impairment scores [61,62,63].

All groups scored high on the preoccupation with Russia-Ukraine War, while they scored lower on changing habits and willingness to change them in the future, although there is a difference between groups. Regarding main fears related to Russia-Ukraine War, adults reported more fear of the rising price of food products and the likelihood of starvation, older adults reported more fear of the rising cost of energy and likelihood of being isolated, while young adults reported more fear of the likelihood of dying and the likelihood of the COVID-19 pandemic not ending or worsening due to war. Future studies could expand the research on age differences in fear of war and even intervene through more specific social policies that can address the individual’s needs. Concerning gender differences, the main difference was found in fear of the likelihood of dying, where women reported more fear in all age groups. This result is coherent with other studies’ findings, where women reported more fear of war [68].

Preoccupation with War is related to depression, anxiety, and stress investigated by DASS in all groups. Fear of war is not related to depression in the young adult and adult groups. Our results support a probable impact on distress about preoccupation with the Russia-Ukraine war in a population not directly involved in the conflict, and the experience of uncertainty of war could have implications for global mental health. Preoccupation with the war at an already sensitive time for the psychological sequelae of COVID could accentuate its long-term effects [71]. Changing habits would seem to be related to anxiety, depression, and stress only in the older adult group; while willingness to change them in the future, would seem to impact stress in all groups. Since this study refers to an Italian sample, it is necessary to consider the long-term economic impact of the conflict between Russia and Ukraine. The price hikes have been difficult for Italy, which is mostly dependent on gas imported from Russia. It is placing upward pressure on inflation at a time when it is already high.

The strengths of the current study consist of examining worries about challenging problems of the 21st century in a wide and large sample of Italian adults; in addition, the research draws attention to new psychological challenges and how these can have a negative influence on well-being. However, the study had some limitations. First, the study design was cross-sectional, which is not very suitable for assessing causality since the temporality of association cannot be checked. The sample had a higher percentage of females than males, which is likely a reflection of how differently the two genders cooperated with the survey, as reported in a similar investigation during the COVD–19 pandemic [34,52,54,55]. The participants were recruited via an online link posted on social networks from central and southern Italy, thus, the generalizability of our findings may be limited compared to a national sample. While online recruitment guarantees large samples, it does not guarantee sample representativeness, as more fragile groups or those who cannot access the Internet were not well represented. The use of specific questions regarding preoccupation, changing habits, and willingness to change habits about 21st-century problems did not enable us to verify the reliability of the responses or to ensure that participants correctly understood the questions.

## 5. Conclusions

In conclusion, the different 21st-century stressors highlighted in this article are associated with individual distress, and it is necessary to consider the possible global mental health problems deriving from these stressors. The results of this specific sample from central and southern Italy are related to the results of national and international research. These challenges should be observed at a global level and addressed with wellness promotion projects at both national and international dimensions. This underlines the need to increase our understanding of the psychological needs of individuals in the 21st century, in which people are under pressure from various problems that impact their quality of life. Different relationships between 21st-century concerns and depression, anxiety, and stress were observed in different age groups; future studies and social policies should consider age differences for an approach tailored to the specific needs of each age group. In addition, new training projects should be developed for mental health workers to better identify and accommodate 21st-century stressors and provide the best psychological support based on the needs and requirements related to these new challenges. Overall, this study highlights areas/risk markers that could benefit from additional research as well as useful information on individual distress in different age groups. Future studies should further investigate the determinants of impaired well-being related to 21st-century concerns, with longer follow-up, and subgroup analyses, following a biopsychosocial approach.

## Figures and Tables

**Table 1:** General characteristics of the enrolled sample (*n =* 1831).

		Young Adults (*n =* 470)	Adults (*n =* 465)	Older Adults (*n =* 460)	Elders (*n =* 436)
Age	Mean	24.18	43.29	55.61	69.47
	Std. Deviation	4.83	3.89	2.63	7.15
	Range	18–35	36–50	51–60	61–93
Gender	Female	348 (74.0%)	282 (60.6%)	226 (49.1%)	260 (59.6%)
	Male	122 (26.0%)	183 (39.4%)	234 (50.9%)	176 (40.4%)
	Total	470 (100%)	465 (100%)	460 (100%)	436 (100%)
Marital Status	Married	31 (6.6%)	272 (58.5%)	191 (41.5%)	245 (56.2%)
	Cohabiting partner	39 (8.3%)	58 (12.5%)	30 (6.5%)	10 (2.3%)
	Separate/Divorced	1 (0.2%)	77 (16.6%)	176 (38.3%)	95 (21.8%)
	Single	195 (41.5%)	34 (7.3%)	30 (6.5%)	14 (3.2%)
	Non-cohabiting partner	204 (43.4%)	18 (3.9%)	20 (4.3%)	9 (2.1%)
	Widow		6 (1.3%)	13 (2.8%)	63 (14.4%)
Children	Yes	42 (8.9%)	328 (70.5%)	270 (58.7%)	330 (75.7%)
	No	428 (91.1%)	137 (29.5%)	190 (41.3%)	106 (24.3%)
Educational Level	Lower than High School	2 (0.4%)	11 (2.4%)	49 (10.7%)	58 (13.3%)
	High School	341 (72.6%)	162 (34.8%)	150 (32.6%)	183 (41.7%)
	Bachelor’s or master’s degree	94 (20.0%)	159 (34.2%)	162 (35.2%)	151 (34.6%)
	Postgraduate degree	33 (7.0%)	133 (28.6%)	99 (21.5%)	45 (10.3%)
Work	Worker	114 (24.3%)	393 (84.5%)	333 (72.4%)	97 (22.2%)
	Retired			15 (3.3%)	215 (49.3%)
	Unemployed	11 (2.3%)	63 (13.5%)	111 (24.1%)	123 (28.2%)
	Student	345 (73.4%)	9 (1.9%)	1 (0.2%)	1 (0.2%)

**Table 2:** Age and gender differences in main fear for COVID-19, Natural Resource Depletion, Russia-Ukrainian war and Climate Change related items.

			Young Adults		Adults		Older Adults		Elders	
			Gender	Total	Gender	Total	Gender	Total	Gender	Total
Main fears for COVID	Possible vaccine consequences	Male	15_x_ (3.6%)	57_a_ (12.1%)	132_x_ (31.7%)	235_b_ (50.5%)	176_x_ (42.3%)	331_c_ (72.0%)	93_x_ (22.4%)	210_b_ (48.2%)
		Female	42_y_ (10.1%)		103_y_ (24.7%)		155_x_ (37.2%)		117_x_ (28.1%)	
	Likelihood of being isolated	Male	36_x_ (70.6%)	145_a_ (30.9%)	4_x_ (7.8%)	47_b_ (10.1%)	1_x_ (2.0%)	10_c_ (2.2%)	10_x_ (19.6%)	21_c_ (4.8%)
		Female	109_x_ (63.4%)		43_y_ (25.0%)		9_x_ (5.2%)		11_y_ (6.4%)	
	Disease and its consequences	Male	71_x_ (28.6%)	268_a_ (57.0%)	47_x_ (19.0%)	183_b_ (39.4%)	57_x_ (23.0%)	119_c_ (25.9%)	73_x_ (29.4%)	205_b_ (47.0%)
		Female	197_y_ (37.4%)		136_y_ (25.8%)		62_y_ (11.8%)		132_x_ (25.0%)	
Main fears for Natural Resource Depletion	Rising prices of food products	Male	37_x_ (11.9%)	139_a_ (29.6)	108_x_ (34.8%)	213_b_ (45.8%)	101_x_ (32.6%)	188_b, c_ (40.9%)	64_x_ (20.6%)	159_a, c_ (36.5%)
		Female	102_y_ (26.2%)		105_y_ (27.0%)		87_y_ (22.4%)		95_x_ (24.4%)	
	Likelihood of starvation	Male	26_x_ (43.3%)	96_a_ (20.4%)	7_x_ (11.7%)	31_b_ (6.7%)	20_x_ (33.3%)	38_b_ (8.3%)	7_x_ (11.7%)	26_b_ (6.0%)
		Female	70_x_ (53.4%)		24_x_ (18.3%)		18_y_ (13.7%)		19_x_ (14.5%)	
	Rising cost of energy	Male	23_x_ (8.6%)	90_a_ (19.1%)	62_x_ (23.0%)	152_b_ (32.7%)	97_x_ (36.1%)	185_b, c_ (40.2%)	87_x_ (32.3%)	209_c_ (47.9%)
		Female	67_y_ (18.3%)		90_x_ (24.5%)		88_y_ (24.0%)		122_x_ (33.2%)	
	Likelihood of dying	Male	36_x_ (47.4%)	145_a_ (39.9%)	6_x_ (7.9%)	69_b_ (14.8%)	16_x_ (21.1%)	49_b_ (10.7%)	18_x_ (23.7%)	42_b_ (9.6%)
		Female	109_x_ (47.6%)		63_y_ (27.5%)		33_x_ (14.4%)		24_y_ (10.5%)	
Main Fears for Russia-Ukrainian war	Rising prices of food products	Male	20_x_ (26.3%)	54_a_ (39.9%)	12_x_ (15.8%)	52_a_ (39.9%)	19_x_ (25.0%)	41_a_ (39.9%)	25_x_ (32.9%)	49_a_ (39.9%)
		Female	34_x_ (28.3%)		40_y_ (33.3%)		22_x_ (18.3%)		24_y_ (20.0%)	
	Likelihood of starvation	Male	5_x_ (6.1%)	17_a_ (3.6%)	27_x_ (32.9%)	50_b_ (10.8%)	27_x_ (32.9%)	42_b_ (9.1%)	23_x_ (28.0%)	44_b_ (10.1%)
		Female	12_y_ (16.9%)		23_x_ (32.4%)		15_x_ (21.1%)		21_x_ (29.6%)	
	Rising cost of energy	Male	37_x_ (16.2%)	92_a_ (19.6%)	58_x_ (25.3%)	113_a, b_ (24.3%)	83_x_ (36.2%)	150_c_ (32.6%)	51_x_ (22.3%)	120_b_. _c_ (27.5%)
		Female	55_x_ (22.4%)		55_x_ (22.4%)		67_y_ (27.2%)		69_x_ (28.0%)	
	Likelihood of being isolated	Male	5_x_ (5.7%)	26_a_ (5.5%)	17_x_ (19.5%)	47_a, b_ (10.1%)	37_x_ (42.5%)	62_b_ (13.5%)	28_x_ (32.2%)	56_b_ (12.8%)
		Female	21_y_ (20.2%)		30_x_ (28.8%)		25_y_ (24.0%)		28_x_ (26.9%)	
	Likelihood of dying	Male	49_x_ (22.2%)	237_a_ (50.4%)	65_x_ (29.4%)	167_b_ (35.9%)	63_x_ (28.5%)	143_b_ (31.1%)	44_x_ (19.9%)	131_b_ (30.0%)
		Female	188_y_ (41.1%)		102_y_ (22.3%)		80_y_ (17.5%)		87_x_ (19.0%)	
	Likelihood that the COVID-19 pandemic will not end or will worsen due to war	Male	6_x_ (30.0%)	44_a_ (9.4%)	4_x_ (20.0%)	36_a, b_ (7.7%)	5_x_ (25.0%)	22_b_ (4.8%)	5_x_ (25.0%)	36_a_. _b_ (8.3%)
		Female	38_x_ (32.2%)		32_x_ (27.1%)		17_x_ (14.4%)		31_x_ (26.3%)	
Main Fears for Climate Change	Likelihood of being isolated	Male	3_x_ (11.5%)	9_a_ (1.9%)	8_x_ (30.8%)	26_b_ (5.6%)	3_x_ (11.5%)	19_a, b_ (4.1%)	12_x_ (46.2%)	28_b_ (6.4%)
		Female	6_x_ (10.7%)		18_x_ (32.1%)		16_x_ (28.6%)		16_x_ (28.6%)	
	Rising prices of food products	Male	23_x_ (5.0%)	67_a_ (14.3%)	139_x_ (30.2%)	244_b_ (52.5%)	188_x_ (40.8%)	325_c_ (70.7%)	111_x_ (24.1%)	274_c_ (62.8%)
		Female	44_y_ (9.8%)		105_y_ (23.4%)		137_y_ (30.5%)		163_y_ (36.3%)	
	Likelihood of starvation	Male	9_x_ (14.8%)	29_a_ (6.2%)	12_x_ (19.7%)	38_a, b_ (8.2%)	18_x_ (29.5%)	35_a, b_ (7.6%)	22_x_ (36.1%)	50_b_ (11.5%)
		Female	20_x_ (22.0%)		26_x_ (28.6%)		17_x_ (18.7%)		28_x_ (30.8%)	
	Likelihood of being forced to leave the place where I live	Male	30_x_ (40.5%)	166_a_ (35.3%)	17_x_ (23.0%)	76_b_ (16.3%)	15_x_ (20.3%)	41_c_ (8.9%)	12_x_ (16.2%)	36_c_ (8.3%)
		Female	136_y_ (55.5%)		59_x_ (24.1%)		26_y_ (10.6%)		24_x_ (9.8%)	
	Likelihood of dying	Male	57_x_ (61.3%)	199_a_ (42.3%)	7_x_ (7.5%)	81_b_ (17.4%)	10_x_ (10.8%)	40_c_ (8.7%)	19_x_ (20.4%)	48_c_ (11.0%)
		Female	142_x_ (51,6%)		74_y_ (26,9%)		30_x_ (10,9%)		29_y_ (10,5%)	

Note. Each same letter in subscript (a-d for age differences and x-y for gender differences) denotes a subset of categories whose proportions (in “total” column for age and in row for gender) do not differ significantly from each other at the 0.05 level (z-test with Bonferroni adjust *p*-values method for multiple comparison).

## Data Availability

All data presented are available upon request from the corresponding author (F.G.).

## Supplementary Materials

The following supporting information can be downloaded at: https://www.mdpi.com/article/10.3390/ijerph191911929/s1.
