# Supplementary material for: Climate Changes, Natural Resources Depletion, COVID-19 Pandemic, and Russian-Ukrainian War: What Is the Impact on Habits Change and Mental Health?
Source: Int J Environ Res Public Health. 2022 Sep 21;19(19):11929. doi: 10.3390/ijerph191911929 (PMC9565033; doi:10.3390/ijerph191911929)
Supplement: Supplementary file 1 [file ijerph-19-11929-s001.zip › ijerph-1874206-supplementary.pdf]

Supplementary Materials

Table S1. Differences in Covid-19, Natural Resource Depletion, Russian-Ukrainian War, and Climate Change preoccupation among age groups controlling for gender

		Mean Adjusted	Std. Deviation	F	df	Sign.
**Covid-19 Preoccupation**	Young Adults	3.04	0.051			
	Adults	2.76	0.051	19.133	3	.000
	Older Adults	2.53	0.052			
	Elders	2.97	0.053			
**Natural Resource Depletion Preoccupation**	Young Adults	3.92	0.057			
	Adults	2.83	0.057	198.911	3	.000
	Older Adults	2.01	0.058			
	Elders	2.42	0.059			
**Russian-Ukrainian War Preoccupation**	Young Adults	4.07	0.041			
	Adults	3.98	0.041	5.462	3	.001
	Older Adults	3.84	0.042			
	Elders	3.98	0.043			
**Climate Change Preoccupation**	Young Adults	3.91	0.060			
	Adults	2.81	0.059	174.150	3	.000
	Older Adults	2.08	0.060			
	Elders	2.44	0.061			

Table S2. Differences in Covid-19, Natural Resource Depletion, Russian-Ukrainian War, and Climate Change habits among age groups controlling for gender

		Mean Adjusted	Std. Deviation	F	df	Sign.
**Covid Change Habits**	Young Adults	3.98	.059			
	Adults	3.39	.058	65.766	3	.000
	Older Adults	3.13	.059			
	Elders	2.85	.060			
**Natural Resource Depletion Change Habits**	Young Adults	3.32	.051			
	Adults	2.74	.051	86.999	3	.000
	Older Adults	2.18	.052			
	Elders	2.49	.053			
**Russian-Ukrainian War Change Habits**	Young Adults	2.21	.041			
	Adults	2.47	.040	29.768	3	.000
	Older Adults	2.63	.041			
	Elders	2.73	.042			
**Climate Change Change Habits**	Young Adults	3.07	.048			
	Adults	2.54	.047	76.265	3	.000
	Older Adults	2.09	.048			
	Elders	2.33	.049			

Table S3. Differences in Covid-19, Natural Resource Depletion, Russian-Ukrainian War, and Climate Change future habits among age groups controlling for gender

		Mean Adjusted	Std. Deviation	F	df	Sign.
**Covid Change Future Habits**	Young Adults	3.44	.061	71.774	3	.000
	Adults	2.58	.061			
	Older Adults	2.28	.062			
	Elders	2.39	.063			
**Natural Resource Depletion Change Future Habits**	Young Adults	4.24	.060	235.976	3	.000
	Adults	2.91	.060			
	Older Adults	2.07	.060			
	Elders	2.55	.062			
**Russian-Ukrainian War Change Future Habits**	Young Adults	3.59	.048	9.829	3	.000
	Adults	3.32	.047			
	Older Adults	3.25	.048			
	Elders	3.32	.049			
**Climate Change Change Future Habits**	Young Adults	4.09	.058	228.468	3	.000
	Adults	2.84	.058			
	Older Adults	2.00	.059			
	Elders	2.49	.060			

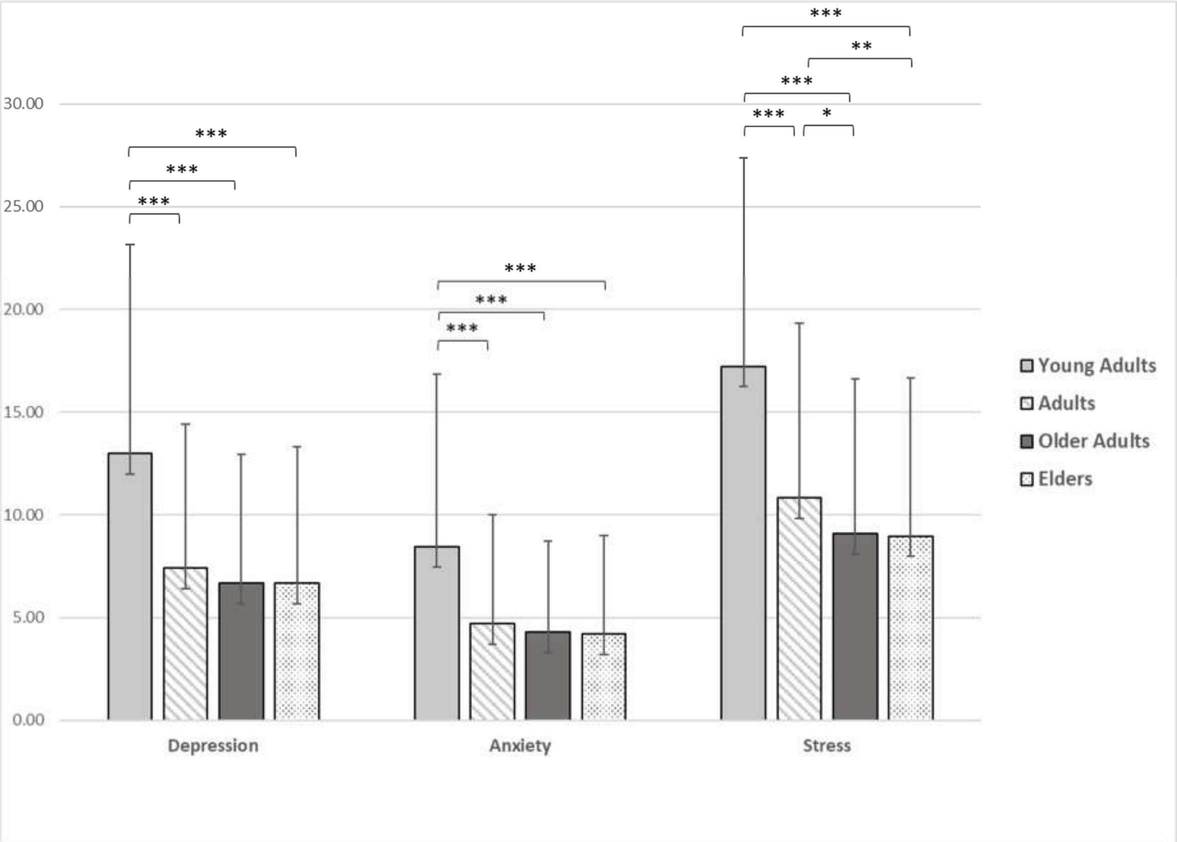

Figure S1. Differences in DASS-21 dimensions among age groups.

Note. * = p < .05, *** = p < .001

Table S4. Differences in DASS-21 dimensions among age groups controlling for gender

		Mean Adjusted	Std. Deviation	F	df	Sign.
**DASS_D**	Young Adults	12.6	.355	62.679	3	.000
	Adults	7.42	.354			
	Older Adults	6.95	.358			
	Elders	6.70	.365			
**DASS_A**	Young Adults	8.20	.274	45.135	3	.000
	Adults	4.71	.273			
	Older Adults	4.55	.276			
	Elders	4.24	.282			
**DASS_S**	Young Adults	16.79	.390	82.311	3	.000
	Adults	10.84	.389			
	Older Adults	9.51	.394			
	Elders	9.06	.401			
